# A proposal for a new staging system for extranodal natural killer T-cell lymphoma: a multicenter study from China and Asia Lymphoma Study Group

**DOI:** 10.1038/s41375-020-0740-1

**Published:** 2020-02-17

**Authors:** Huangming Hong, Yexiong Li, Soon Thye Lim, Chaoyong Liang, He Huang, Pingyong Yi, Tao Wu, Xin Du, Mingzhi Zhang, Jinghua Wang, Jun Zhu, Ting Liu, Fanyi Meng, Gang Wu, Ye Guo, Yuan Zhu, Weili Zhao, Jie Jin, Juan Li, Yanming Deng, Kangsheng Gu, Xiangyuan Wu, Xiaoyan Ke, Derong Xie, Daren Lin, Zhigang Peng, Junxin Wu, Qing Liu, Won Seog Kim, Tongyu Lin

**Affiliations:** 1 Department of Medical Oncology, Sun Yat-sen University Cancer Center, State Key Laboratory of Oncology in Southern China, and Collaborative Innovation Center of Cancer Medicine, Guangzhou, China; 2 Department of Medical Oncology, Sun Yat-sen Memorial Hospital, Sun Yat-Sen University, Guangzhou, China; 3 Department of Radiation Oncology, Cancer Institute and Hospital, Chinese Academy of Medical Sciences, Beijing, China; 4 Division of Medical Oncology, National Cancer Centre Singapore, Singapore, Singapore; 5 Department of Medical Oncology, Guangxi Medical University Cancer Hospital, Nanning, China; 6 Department of Medical Oncology, Hunan Cancer Hospital, Changsha, China; 7 Department of Lymphoma-Oncology, Guizhou Cancer Hospital, Guiyang, Guizhou China; 8 Department of Hematology, Guangdong General Hospital, Guangzhou, China; 9 Department of Medical Oncology, the First Affiliated Hospital of Zhengzhou University, Zhengzhou, China; 10 Department of Medical Oncology, Nanjing General Hospital of Nanjing Military Command, Nanjing, China; 11 Department of Lymphoma-Oncology, Peking University Cancer Hospital, Beijing, China; 12 Department of Hematology, West China Hospital of Sichuan University, Chengdu, China; 13 Department of Hematology, Nanfang Hospital of Southern Medical University, Guangzhou, China; 14 Department of Radiation Oncology, Wuhan Union Hospital, Wuhan, China; 15 Department of Medical Oncology, Shanghai East Hospital, Tongji University School of Medicine, Shanghai, China; 16 Department of Radiation Oncology, Zhejiang Cancer Hospital, Hangzhou, China; 17 Department of Hematology, Ruijin Hospital, Shanghai Jiao Tong University School of Medicine, Shanghai, China; 18 Department of Hematology, the First Affiliated Hospital Zhejiang University, Hangzhou, China; 19 Department of Hematology, the First Affiliated Hospital of Sun Yat-sen University, Guangzhou, China; 20 Department of Medical Oncology, the First People’s Hospital of Foshan, Foshan, China; 21 Department of Medical Oncology, the First Affiliated Hospital of Anhui Medical University, Hefei, China; 22 Department of Medical Oncology, the Third Affiliated Hospital of Sun Yat-sen University, Guangzhou, China; 23 Department of Hematology, Peking University Third Hospital, Beijing, China; 24 Department of Medical Oncology, Jiangmen Central Hospital, Jiangmen, China; 25 Department of Medical Oncology, the First Affiliated Hospital of Guangxi Medical University, Nanning, China; 26 Department of Radiation Oncology, Fujian Provincial Cancer Hospital, Fuzhou, Fujian, China; 27 Department of Epidemiology, Sun Yat-sen University Cancer Center, Guangzhou, China; 28 Division of Hematology Oncology, Department of Medicine, Samsung Medical Center, Sungkyunkwan University School of Medicine, Seoul, Korea

**Keywords:** Risk factors, T-cell lymphoma

## To the Editor:

Extranodal natural killer T-cell lymphoma (ENKTL) is a distinct entity in the World Health Organization (WHO) classification. Patients suffering from this type of disease have poor survival outcomes [1–7]. However, using the Ann Arbor staging system (AASS), a routine lymphoma staging system, most ENKTL patients are categorized as early stage, which is inconsistent with their poor survival [1, 8–12]. Since the AASS has limited utility in the prognostication and treatment decision making for patients with ENKTL, this study aimed to develop a new staging system specific for ENKTL that can effectively identify patients with poor prognosis and provide information for personalized therapy.

There were three components of this study: a training cohort consisting of two stages and a validation cohort. In the training cohort, we first conducted a retrospective study of ENKTL patients treated with cyclophosphamide, doxorubicin, vincristine, and prednisolone (CHOP) or CHOP-like regimens or radiotherapy (RT) alone between Jan 1999 and Jun 2008 in 19 hospitals in China, with the aim to identify high-risk factors for proposing the new staging system: the Chinese Southwest Oncology Group and Asia Lymphoma Study Group ENKTL (CA) system. Second, we conducted a prospective study of patients treated with asparaginase-based regimens or RT between Jul 2008 and Dec 2012 in the same 19 hospitals to determine whether the CA system was suitable in the era of asparaginase-based treatment. Based on the results of retrospective study and prospective study, the final form of the CA system was established. To validate the results from the training cohort, we performed an independent validation cohort study between Jan 2010 and Dec 2017 with the same inclusion and exclusion criteria using data obtained from Samsung Medical Center in South Korea, the National Cancer Center in Singapore and five hospitals in China, that were not included in the training cohort. The inclusion criteria were: (1) central pathologically confirmed diagnosis of ENKTL according to the 2008 WHO classification of lymphomas; (2) treatment with chemotherapy with or without radiotherapy or with RT alone with curative intent; and (3) availability of all clinical data required for staging and survival analyses. Local invasiveness was defined as invasion of the bone or perforation or invasion of the skin or paranasal extension as previously reported [9]. Regional lymph node involvement was defined as the invasion of lymph nodes corresponding to N1, N2, or N3 of the primary lesion in accordance with 2002 TNM classification of the American Joint Committee on Cancer. The nasal and nonnasal types were defined based on the involvement of the nasal area, as reported previously [5]. The Institutional Review Board of Sun Yat-sen University Cancer Center (Guangzhou, China) reviewed and approved all aspects of this study. The statistical methods are summarized in Supplementary Appendix.

The CONSORT flow of the study was shown in Supplementary Fig. 1. The training cohort included 1168 patients. Table 1 summarizes the characteristics of the patients. In the retrospective study, patients with lesions confined to the nasal cavity or nasopharynx without local tumor invasiveness showed a superior 5-year overall survival (OS) rate than patients with lesions complicated by local tumor invasiveness (59.7% vs. 48.1%, *P* = 0.01). Those with nonnasal-type disease without lymph node involvement had a lower 5-year OS rate than those with nasal-type disease without lymph node involvement (56.7% vs. 40.1%, *P* < 0.01). Thus, patients with nonnasal-type disease or lesions confined to the nasal cavity or nasopharynx complicated by local tumor invasiveness were classified as stage II. Patients harboring lesions with regional lymph node involvement exhibited a lower 5-year OS rate than those without regional lymph node involvement (34.7% vs. 52.3%, *P* < 0.01). Thus, patients harboring lesions with regional lymph node involvement were categorized as stage III. Further, patients with non-regional lymph node involvement or lymph node involvement on both sides of the diaphragm or disseminated disease did not show 5-year OS rate difference (26.5% vs. 27.1% vs. 25.7%, *P* = 0.342), these patients were classified as stage IV. Thus, we propose that the CA be stratified as follows: stage I, lesions confined to the nasal cavity or nasopharynx without local invasiveness and lymph node involvement; stage II, nonnasal-type disease or lesions confined to the nasal cavity or nasopharynx with local invasiveness without lymph node involvement; stage III, lesions with regional lymph node involvement; and stage IV, involvement of nonregional lymph node or lymph nodes on both sides of the diaphragm or disseminated disease. In the retrospective study involving patients who received CHOP-like treatment, the CA effectively discriminated survival; we then performed the prospective study and found that CA also effectively in discriminating the survival of patients who received asparaginase-based treatment.

**Table 1:** Comparison of characteristics between the validation and training cohorts.

Characteristic	Training cohort *n* = 1168, no. (%)	Validation cohort *n* = 985, no. (%)	*P*
Age, years			0.719
≤60	985 (84.3)	837 (85.0)	
>60	183 (15.7)	148 (15.0)	
Sex			0.166
Male	804 (68.8)	650 (66.0)	
Female	364 (31.2)	335 (34.0)	
ECOG PS			0.237
0–1	1061 (90.8)	896 (91.0)	
2–4	87 (9.2)	89 (9.0)	
“B” symptoms			0.242
Absent	570 (48.8)	455 (46.2)	
Present	598 (51.2)	530 (53.8)	
Serum LDH level			0.510
Normal	822 (70.5)	680 (69.0)	
Elevated	346 (29.6)	305 (31.0)	
IPI			0.377
0–1	869 (74.4)	716 (72.7)	
2–5	299 (25.6)	269 (27.3)	
NK prognostic index			0.189
1–2	690 (59.1)	554 (56.2)	
3–4	478 (40.9)	431 (43.8)	
Local invasiveness			0.405
Absent	688 (58.9)	562 (57.1)	
Present	480 (41.1)	423(42.9)	
Nonnasal type			0.498
Yes	162 (13.9)	147 (14.9)	
No	1006 (86.1)	838 (85.1)	
Regional lymph-node involvement			0.500
Absent	950 (81.3)	813 (82.5)	
Present	218 (18.7)	172 (17.5)	
Bone marrow involvement			0.441
Absent	1093 (93.6)	913 (92.7)	
Present	75 (6.4)	72 (7.3)	
Ann Arbor staging system stage			0.492
I–II	961 (82.3)	822 (83.5)	
III–IV	207 (17.7)	163 (16.5)	
WBC count			0.232
>4000 per mm^3^	954 (81.7)	824 (83.7)	
<4000 per mm^3^	214 (18.3)	161 (16.3)	
Hb level			0.869
>110 g/L	945 (80.9)	800 (81.2)	
<110 g/L	223 (19.1)	185 (18.8)	
Platelet count			0.877
>100,000 per mm^3^	1070 (91.6)	900 (91.4)	
<100,000 per mm^3^	98 (8.4)	85 (8.6)	
Absolute lymphocyte count			0.165
>1000 per mm^3^	808 (69.2)	653 (66.3)	
<1000 per mm^3^	360 (30.8)	332 (33.7)	
Serum albumin level			0.240
>35 g/L	965 (82.6)	794 (80.6)	
<35 g/L	203 (17.4)	191 (19.4)	
Treatment regimens			
Anthracycline-based			<0.001
RT alone	200 (23.1)	20 (4.5)	
CHOP	502 (58.1)	318 (71.1)	
CHOP-like	162 (18.8)	109 (24.4)	
Asparaginase-based			0.070
RT alone	82 (30.0)	186 (34.6)	
SMILE-like	49 (16.1)	73 (13.6)	
Platinum containing	173 (56.9)	279 (51.8)	

Local invasiveness was defined in the text.

*ECOG PS* eastern cooperative oncology group performance status, *LDH* lactate dehydrogenase, *IPI* International Prognostic Index, *NK* natural killer, *WBC* white blood cell, *Hb* hemoglobin, *RT* radiotherapy, *CHOP* cyclophosphamide, doxorubicin, vincristine, and prednisolone, *SMILE* dexamethasone, methotrexate, ifosfamide, l-asparaginase, and etoposide.

According to the AASS, the patient distribution in the training cohort from stages I through IV was 61.8%, 20.4%, 5.7%, and 12.1%, respectively. However, according to the CA system, the distribution was 27.4%, 35.2%, 18.7%, and 18.7%, respectively, from stages I through IV (Supplementary Table 1).

**Fig. 1:**
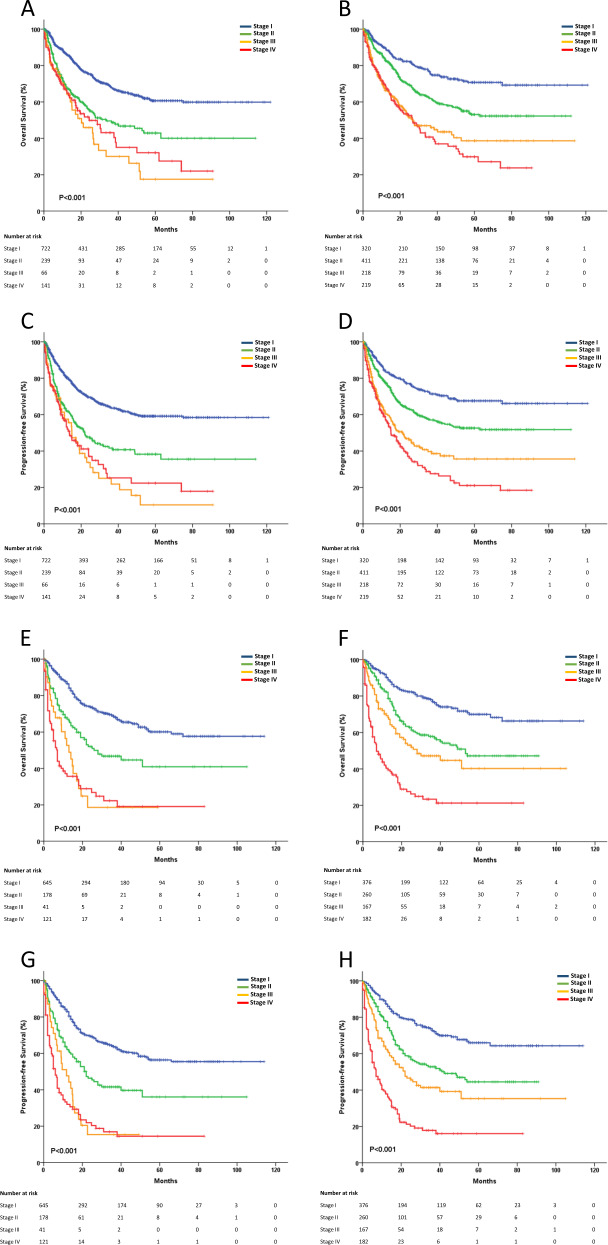
The OS and PFS of the training cohort and the validation cohort. **a** OS staging using the AASS for the training cohort. **b** OS staging using the CA system for the training cohort. **c** PFS staging using the AASS for the training cohort. **d** PFS staging using the CA system for the training cohort. **e** OS staging using the AASS for the validation cohort. **f** OS staging using the CA system for the validation cohort. **g** PFS staging using the AASS for the validation cohort. **h** PFS staging using the CA system for the validation cohort.

The 5-year OS rate for the training cohort was 52.4% (95% confidence interval (CI) 48.9–55.9), and the 5-year progression-free survival (PFS) rate was 49.0% (95% CI 45.5–52.5). In the training cohort, the CA system exhibited good patient stratification, with 5-year OS rates of 70.8%, 53.1%, 38.6%, and 29.9% for stages I through IV (*P* < 0.001), respectively, and 5-year PFS rates of 67.5%, 52.6%, 35.6%, and 21.1% (*P* < 0.001), respectively. Alternatively, the 5-year OS rates were 60.7%, 42.9%, 17.5%, and 32.1% for AASS stages I through IV (*P* < 0.001), respectively, and the 5-year PFS rates were 59.1%, 38.3%, 10.4%, and 22.4% (*P* < 0.001), respectively (Fig. 1a–d). For patients receiving CHOP-like treatment who were diagnosed with CA stages I through IV, the 5-year OS rates also showed reasonable declines; however, when diagnosed using the AASS, the survival of staging IV was better than that of staging III (Supplementary Fig. 2a, b). This result was similar for patients receiving asparaginase-based treatment (Supplementary Fig. 2c, d).

Patients from Singapore (*n* = 114), South Korea (*n* = 102), and China (*n* = 769) were included in the independent validation cohort, the 5-year OS rate was 50.4% (95% CI 46.1–54.7), and the 5-year PFS rate was 46.0% (95% CI 41.7–50.3) in this cohort. The CA system effectively stratified the OS and PFS for all 985 patients. (Fig. 1e–h).

In the receiver operating characteristic (ROC) analysis, the CA system better discriminated survival than the AASS in the training cohort (area under the curve (AUC), 0.68 vs. 0.60, *P* = 0.013) and the validation cohort (AUC, 0.70 vs. 0.61, *P* = 0.032). For all 842 patients who received asparaginase-based treatment in the training cohort and validation cohort, the prognostic index of natural killer lymphoma (PINK) [13] could stratify the survival according to different risk groups (Supplementary Fig. 3). However, the ROC analysis of CA system was superior than PINK (AUC, 0.71 vs. 0.64, *P* = 0.031). For the 842 asparaginase-based treatment patients, for CA stage I, the 5-year OS rates for RT and chemotherapy combined with radiotherapy were similar (81.6% vs. 85.8%, *P* = 0.248). For CA stage II, RT resulted in the lowest 5-year OS rate of 70.1%, while concurrent chemoradiotherapy (CCRT), induction chemotherapy followed by radiotherapy, or concurrent chemotherapy (CT + CCRT/RT) and CCRT followed by adjuvant chemotherapy (CCRT + CT) showed similar 5-year OS rates (75.2%, 82.3%, and 76.7%, respectively, *P* = 0.754). Patients with CA stage III receiving CT + CCRT/RT exhibited the highest 5-year OS rate of 73.5%, CCRT + CT and CCRT had moderate 5-year OS rates of 67.0% and 55.3%, respectively, and those receiving RT had the lowest 5-year OS of 32.3% (*P* < 0.001). For CA stage IV, patients receiving autologous transplantation after chemotherapy did not show superior survival than those who did not (57.1% vs. 23.5%, *P* = 0.174) (Supplementary Fig. 4).

The AASS could not reasonably stratify the survival of ENKTL patients, since the survival of patients with stage IV was better than that of stage III. Less than 10% of patients were classified as stage III by the AASS, the highly unbalanced distribution may produce unavoidable survival bias. Yan et al. [14] recently suggested a TNM staging system for ENKTL. However, that study was performed at a single-center, focused only on nasal patients, and the majority of patients enrolled received anthracycline chemotherapy, thus limiting the generalizability of that staging system. Currently, PINK is used to predict prognosis, but factors including the stage and lymph node involvement in this index are traditionally classified as part of the staging system. Thus, the application of PINK should depending on patient’s general characteristics and staging factors. The CA system was established based on anatomic factors and can efficiently classify patients into different stages. The anatomic factors can be conveniently examined through imaging examinations. Moreover, the ROC analysis suggested that CA staging system is superior to AASS and PINK.

In terms of guiding contemporary asparaginase-based treatment, our study recommended RT for stage I; chemotherapy combined with radiotherapy for stage II; CT + CCRT/RT for stage III; and intensive chemotherapy for stage IV. However, the treatment regimens in our study were varied, validation in a second data set is needed, most preferable in a prospective manner.

The CA system demonstrated better survival discrimination than the AASS, and might add prognostic value and inform treatment decisions for ENKTL. It is crucial to accurately identify high-risk patients to improve outcomes in this subset of lymphoma.

### Supplementary information

Statistical analysis

Patient distribution according to the different staging systems

Supplementary Figures Legends

Supplementary Figure 1–4
