# Supplementary material for: A proposal for a new staging system for extranodal natural killer T-cell lymphoma: a multicenter study from China and Asia Lymphoma Study Group
Source: Leukemia. 2020 Feb 17;34(8):2243–8. doi: 10.1038/s41375-020-0740-1 (PMC7387308; doi:10.1038/s41375-020-0740-1)
Supplement: Supplementary file 1 — Statistical analysis [file 41375_2020_740_MOESM1_ESM.docx]

***Statistical analysis***

We compared overall survival (OS) outcomes according to all pretreatment characteristics; the study period was defined as the time from the initial diagnosis to the last follow-up date or the date of death by any cause. If patients were alive at the time of data analysis, they were censored at the date of their last follow-up visit. We also compared progression-free survival (PFS) outcomes in our new staging system. We defined progression-free survival as the time from the initial diagnosis to the date of relapse, progression, last follow-up, or any type of death.

We used survival analysis-related methods to develop the CA system. Based on the survival curve estimated by the Kaplan-Meier method, the log-rank test was used to identify high-risk factors for ENKTL. The chi-square test was applied to identify associations between categorical variables. We used receiver operating characteristic (ROC) curves to compare the sensitivity and specificity for predicting whether or not patients survived for at least five years. Patients who did not die but with observation time less than 5 years have been excluded from this analysis. P values less than 0.05 were considered statistically significant, and all P values were calculated via two-sided significance tests. Statistical analyses were performed using SPSS 17.0.
