# Supplementary material for: A proposal for a new staging system for extranodal natural killer T-cell lymphoma: a multicenter study from China and Asia Lymphoma Study Group
Source: Leukemia. 2020 Feb 17;34(8):2243–8. doi: 10.1038/s41375-020-0740-1 (PMC7387308; doi:10.1038/s41375-020-0740-1)
Supplement: Supplementary file 2 — Patient distribution according to the different staging systems [file 41375_2020_740_MOESM2_ESM.docx]

| **Supplementary Table 1.** Patient distribution according to the different staging systems | | | | |
| --- | --- | --- | --- | --- |
|  | Training cohort | | Validation cohort | |
|  | CA stage,  No. (%) | AASS stage,  No. (%) | CA stage,  No. (%) | AASS stage,  No. (%) |
| Stage I | 320 (27.4) | 722 (61.8) | 376 (38.2) | 645 (65.5) |
| Stage II | 411 (35.2) | 239 (20.4) | 260 (26.4) | 178 (18.1) |
| Stage III | 218 (18.7) | 66 (5.7) | 167 (17.0) | 41 (4.2) |
| Stage IV | 219 (18.7) | 141 (12.1) | 182 (18.5) | 121 (12.3) |
| CA, Chinese Southwest Oncology Group (CSWOG) and Asia Lymphoma Study Group (ALSG) ENKTL (CA) system; AASS, Ann Arbor staging system | | | | |
