# Supplementary material for: A proposal for a new staging system for extranodal natural killer T-cell lymphoma: a multicenter study from China and Asia Lymphoma Study Group
Source: Leukemia. 2020 Feb 17;34(8):2243–8. doi: 10.1038/s41375-020-0740-1 (PMC7387308; doi:10.1038/s41375-020-0740-1)
Supplement: Supplementary file 3 — Supplementary Figures Legends [file 41375_2020_740_MOESM3_ESM.docx]

**Supplementary Figures**

**Supplementary Figure 1.** CONSORT diagram. (A) The training cohort and (B) The validation cohort.

**Supplemental Figure 2.** The OS of the retrospective and prospective groups. (A) Retrospective group staging using the AASS. (B) Retrospective group staging using the CA system. (C) Prospective group staging using the AASS. (D) Prospective group staging using the CA system.

**Supplementary Figure 3.** The OS of the patients who received asparaginase-based treatment. (A) Patients were stratified according to the PINK indices. (B) Patients were stratified according to the CA system.

**Supplementary Figure 4.** Subgroup analysis of asparaginase-based treatment in the CA. (A) Stage I patients; (B) Stage II patients; (C) Stage III patients; and (D) Stage IV patients.
