# Supplementary material for: A proposal for a new staging system for extranodal natural killer T-cell lymphoma: a multicenter study from China and Asia Lymphoma Study Group
Source: Leukemia. 2020 Feb 17;34(8):2243–8. doi: 10.1038/s41375-020-0740-1 (PMC7387308; doi:10.1038/s41375-020-0740-1)
Supplement: Supplementary file 4 — Supplementary Figure 1–4 [file 41375_2020_740_MOESM4_ESM.pptx]

## Slide 1
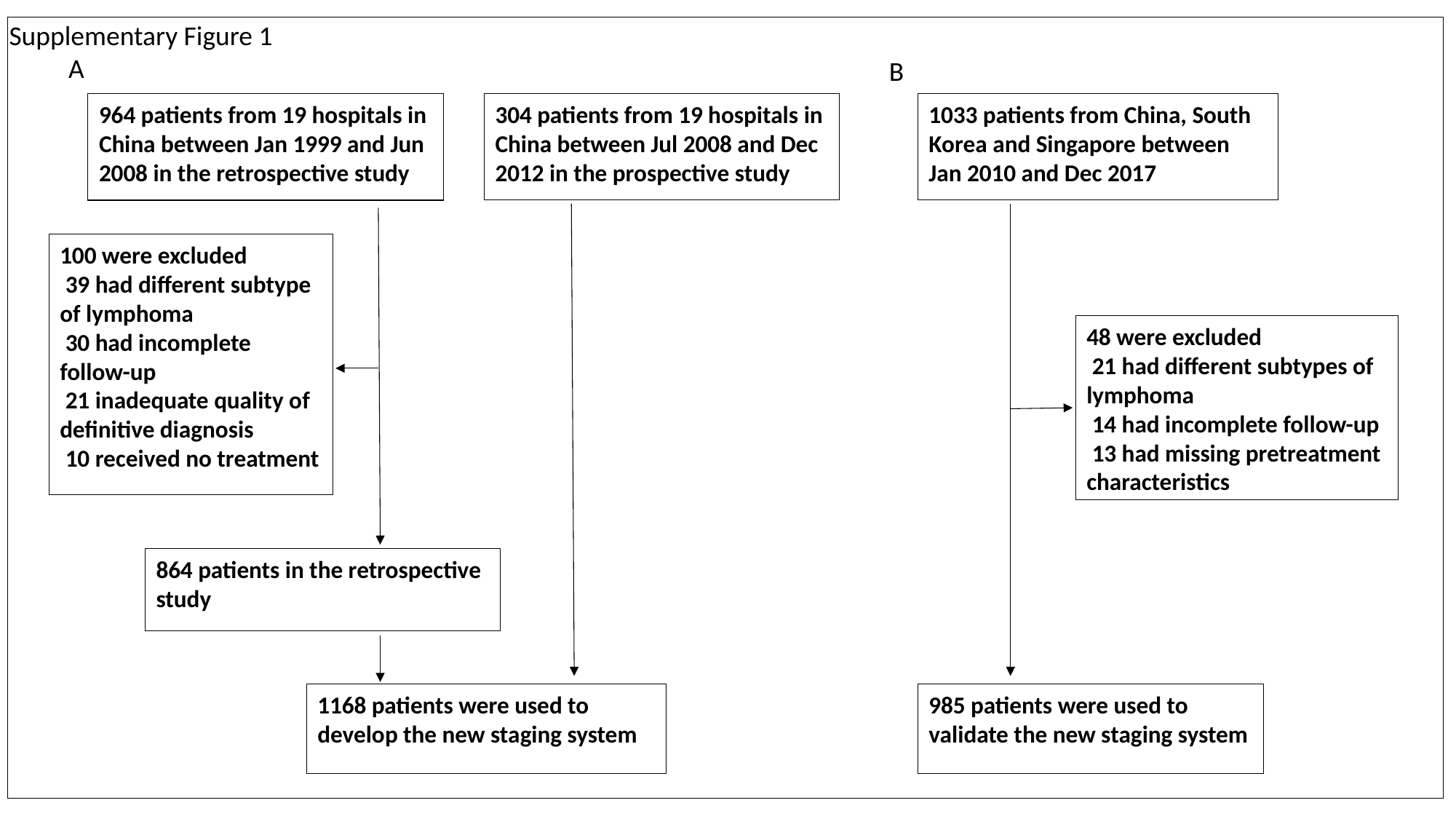

Supplementary Figure 1
A
B
304 patients from 19 hospitals in China between Jul 2008 and Dec 2012 in the prospective study
964 patients from 19 hospitals in China between Jan 1999 and Jun 2008 in the retrospective study
1033 patients from China, South Korea and Singapore between Jan 2010 and Dec 2017
100 were excluded
 39 had different subtype of lymphoma
 30 had incomplete follow-up
 21 inadequate quality of definitive diagnosis
 10 received no treatment
48 were excluded
 21 had different subtypes of lymphoma
 14 had incomplete follow-up
 13 had missing pretreatment
characteristics
1168 patients were used to develop the new staging system
985 patients were used to validate the new staging system
864 patients in the retrospective study

## Slide 2
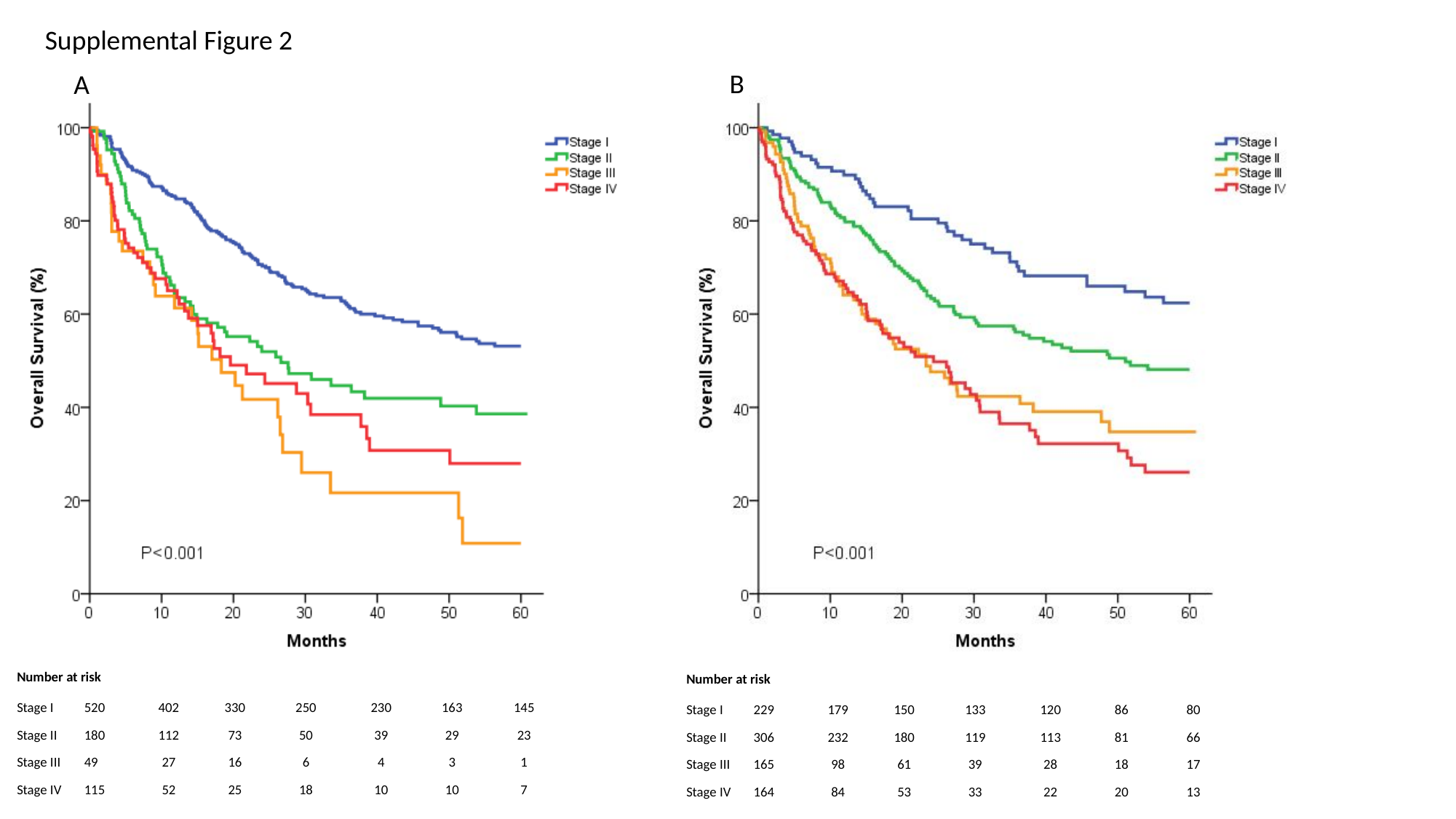

Supplemental Figure 2
B
A
| Number at risk | | | | | | | |
| --- | --- | --- | --- | --- | --- | --- | --- |
| Stage I | 520 | 402 | 330 | 250 | 230 | 163 | 145 |
| Stage II | 180 | 112 | 73 | 50 | 39 | 29 | 23 |
| Stage III | 49 | 27 | 16 | 6 | 4 | 3 | 1 |
| Stage IV | 115 | 52 | 25 | 18 | 10 | 10 | 7 |
| Number at risk | | | | | | | |
| --- | --- | --- | --- | --- | --- | --- | --- |
| Stage I | 229 | 179 | 150 | 133 | 120 | 86 | 80 |
| Stage II | 306 | 232 | 180 | 119 | 113 | 81 | 66 |
| Stage III | 165 | 98 | 61 | 39 | 28 | 18 | 17 |
| Stage IV | 164 | 84 | 53 | 33 | 22 | 20 | 13 |

## Slide 3
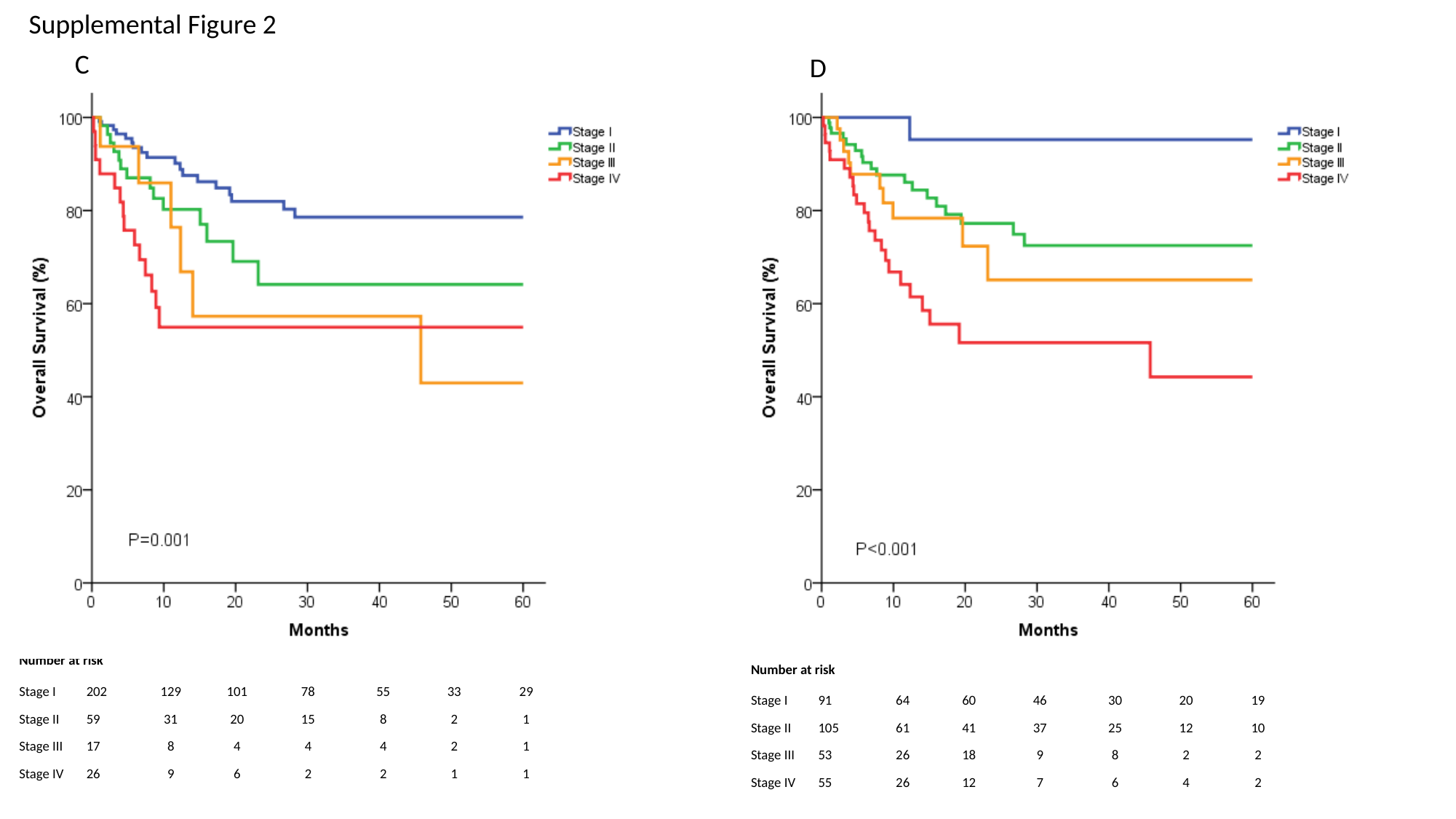

Supplemental Figure 2
C
D
| Number at risk | | | | | | | |
| --- | --- | --- | --- | --- | --- | --- | --- |
| Stage I | 202 | 129 | 101 | 78 | 55 | 33 | 29 |
| Stage II | 59 | 31 | 20 | 15 | 8 | 2 | 1 |
| Stage III | 17 | 8 | 4 | 4 | 4 | 2 | 1 |
| Stage IV | 26 | 9 | 6 | 2 | 2 | 1 | 1 |
| Number at risk | | | | | | | |
| --- | --- | --- | --- | --- | --- | --- | --- |
| Stage I | 91 | 64 | 60 | 46 | 30 | 20 | 19 |
| Stage II | 105 | 61 | 41 | 37 | 25 | 12 | 10 |
| Stage III | 53 | 26 | 18 | 9 | 8 | 2 | 2 |
| Stage IV | 55 | 26 | 12 | 7 | 6 | 4 | 2 |

## Slide 4
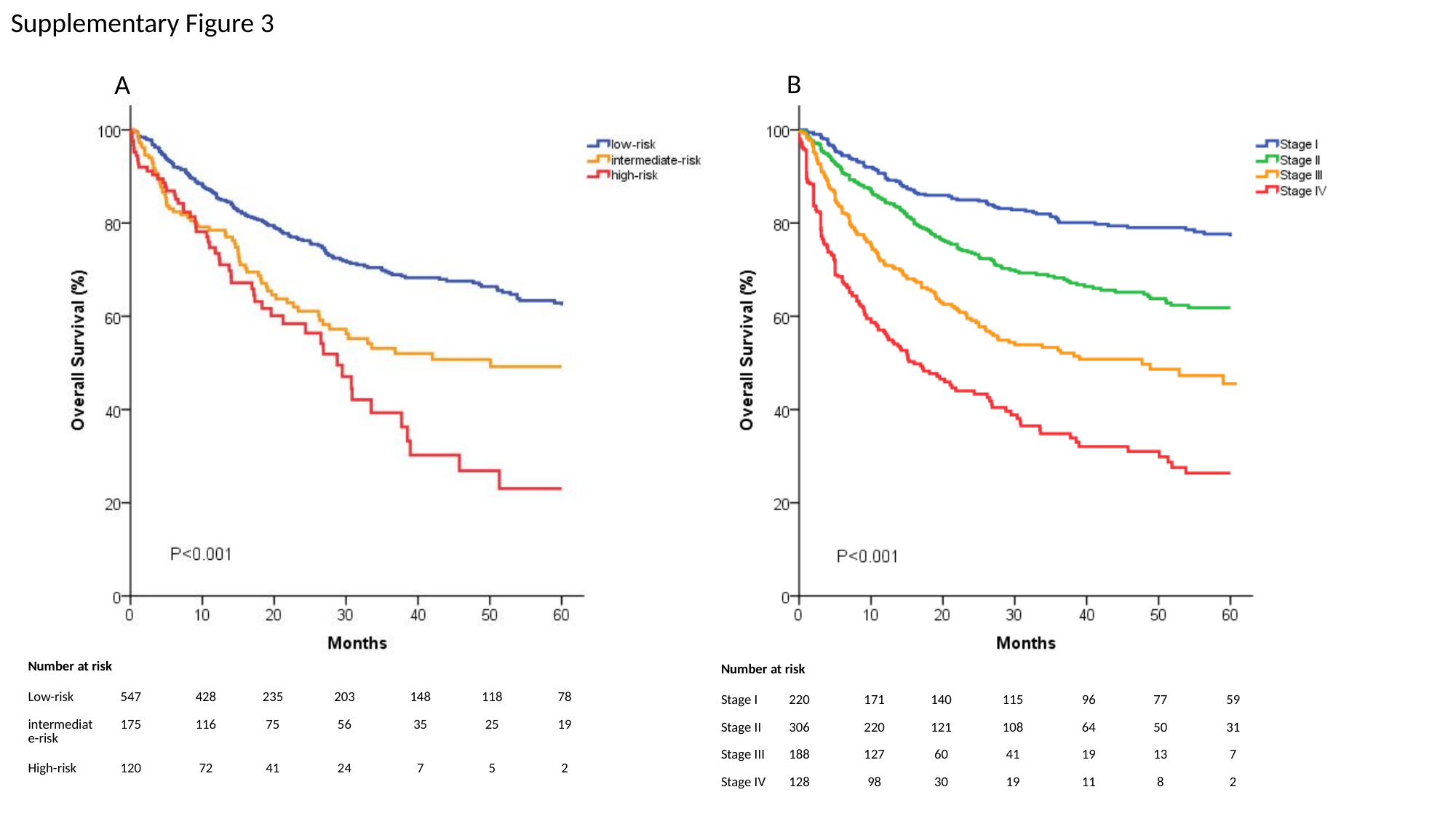

Supplementary Figure 3
B
A
| Number at risk | | | | | | | |
| --- | --- | --- | --- | --- | --- | --- | --- |
| Low-risk | 547 | 428 | 235 | 203 | 148 | 118 | 78 |
| intermediate-risk | 175 | 116 | 75 | 56 | 35 | 25 | 19 |
| High-risk | 120 | 72 | 41 | 24 | 7 | 5 | 2 |
| Number at risk | | | | | | | |
| --- | --- | --- | --- | --- | --- | --- | --- |
| Stage I | 220 | 171 | 140 | 115 | 96 | 77 | 59 |
| Stage II | 306 | 220 | 121 | 108 | 64 | 50 | 31 |
| Stage III | 188 | 127 | 60 | 41 | 19 | 13 | 7 |
| Stage IV | 128 | 98 | 30 | 19 | 11 | 8 | 2 |

## Slide 5
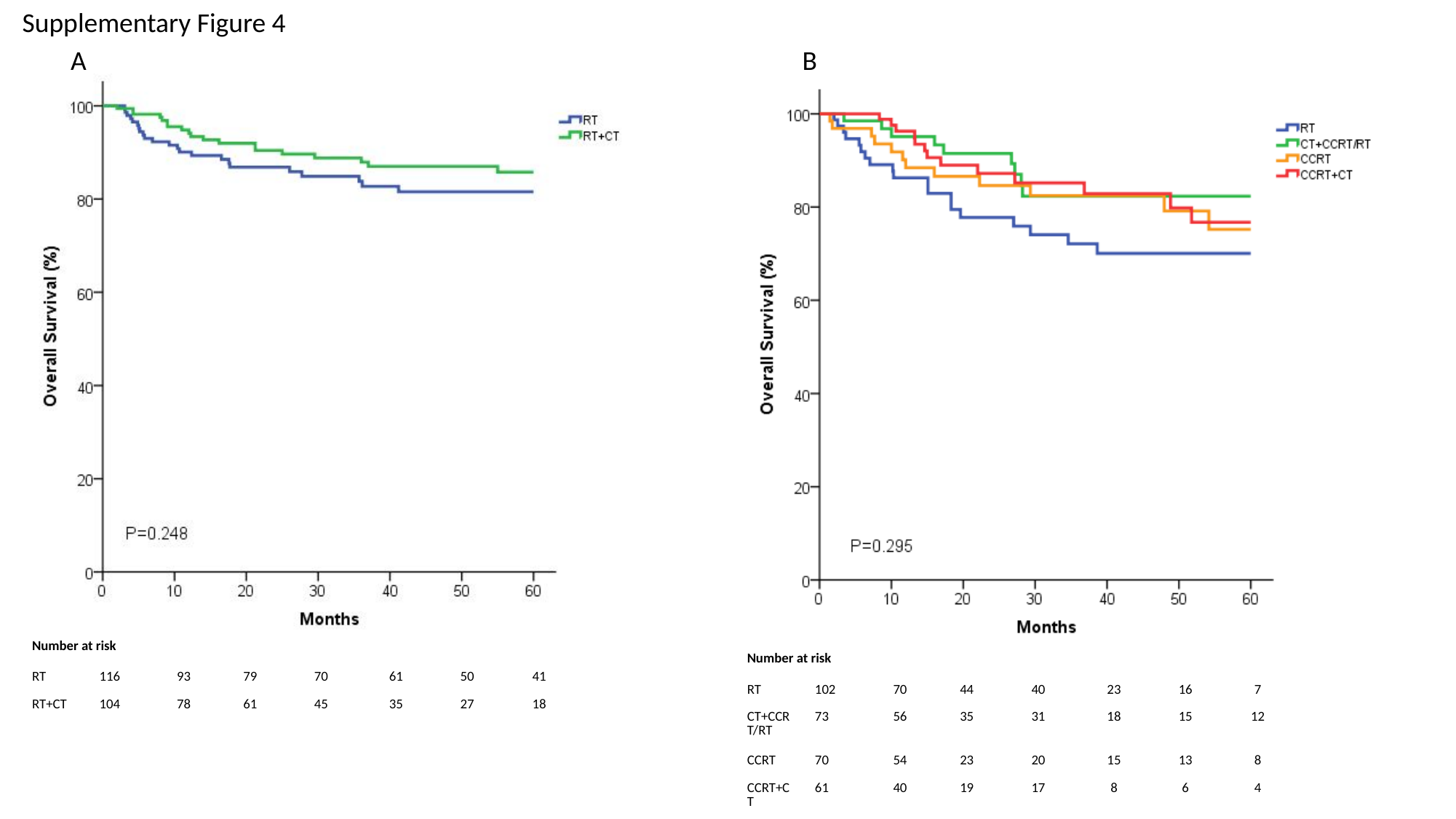

Supplementary Figure 4
A
B
| Number at risk | | | | | | | |
| --- | --- | --- | --- | --- | --- | --- | --- |
| RT | 116 | 93 | 79 | 70 | 61 | 50 | 41 |
| RT+CT | 104 | 78 | 61 | 45 | 35 | 27 | 18 |
| Number at risk | | | | | | | |
| --- | --- | --- | --- | --- | --- | --- | --- |
| RT | 102 | 70 | 44 | 40 | 23 | 16 | 7 |
| CT+CCRT/RT | 73 | 56 | 35 | 31 | 18 | 15 | 12 |
| CCRT | 70 | 54 | 23 | 20 | 15 | 13 | 8 |
| CCRT+CT | 61 | 40 | 19 | 17 | 8 | 6 | 4 |

## Slide 6
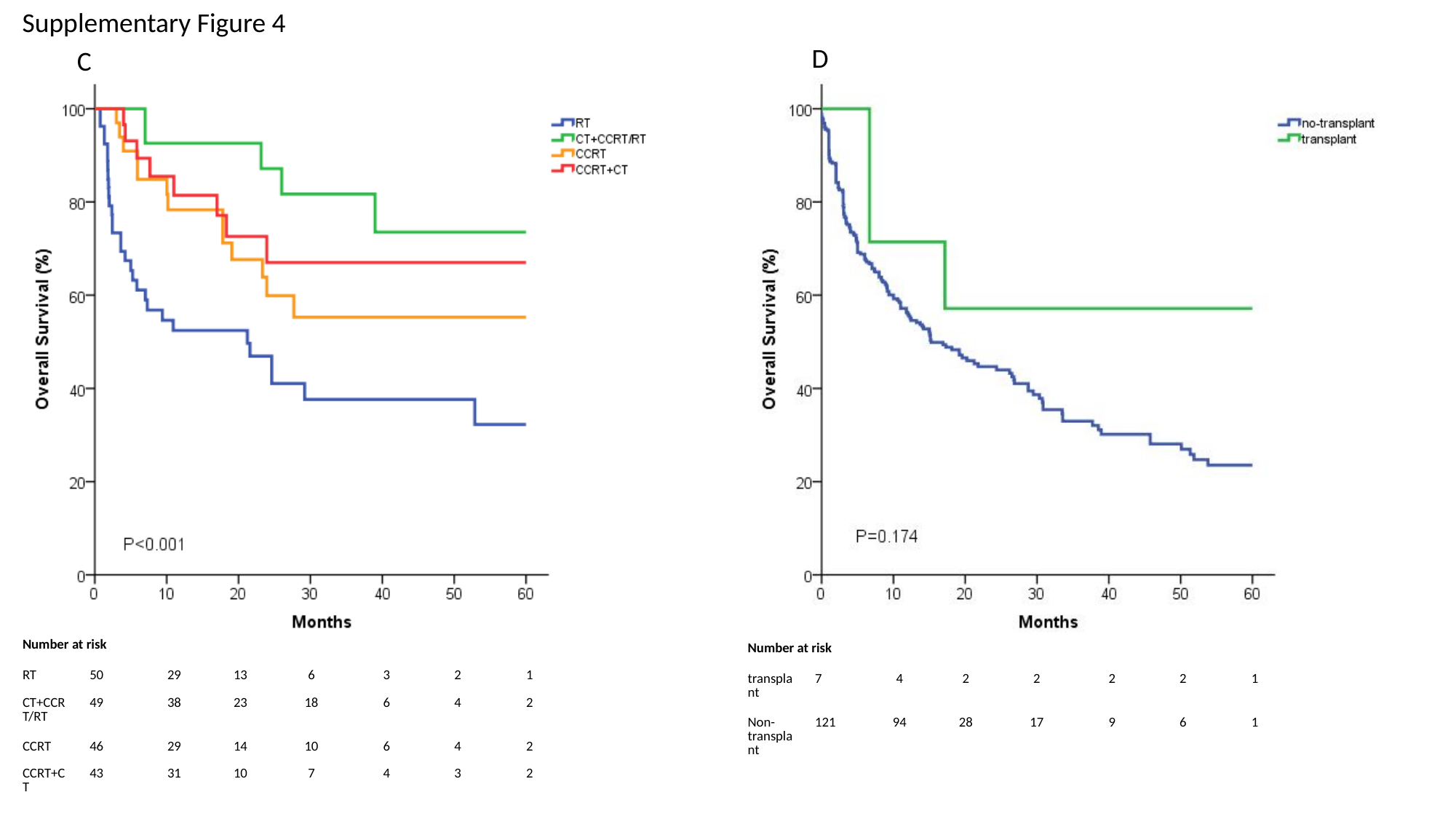

Supplementary Figure 4
D
C
| Number at risk | | | | | | | |
| --- | --- | --- | --- | --- | --- | --- | --- |
| RT | 50 | 29 | 13 | 6 | 3 | 2 | 1 |
| CT+CCRT/RT | 49 | 38 | 23 | 18 | 6 | 4 | 2 |
| CCRT | 46 | 29 | 14 | 10 | 6 | 4 | 2 |
| CCRT+CT | 43 | 31 | 10 | 7 | 4 | 3 | 2 |
| Number at risk | | | | | | | |
| --- | --- | --- | --- | --- | --- | --- | --- |
| transplant | 7 | 4 | 2 | 2 | 2 | 2 | 1 |
| Non-transplant | 121 | 94 | 28 | 17 | 9 | 6 | 1 |
